# Benthic Peracarids (Crustacea) from an unexplored area of Patagonian channels and Fjords

**DOI:** 10.3897/BDJ.8.e58013

**Published:** 2020-09-28

**Authors:** Patricia Esquete, Cristian Aldea

**Affiliations:** 1 CESAM & Departamento de Biologia. Universidade de Aveiro, Aveiro, Portugal CESAM & Departamento de Biologia. Universidade de Aveiro Aveiro Portugal; 2 GAIA Antarctic Research Center (CIGA) and Department of Science and Natural Resources. Universidad de Magallanes, Punta Arenas, Chile GAIA Antarctic Research Center (CIGA) and Department of Science and Natural Resources. Universidad de Magallanes Punta Arenas Chile

**Keywords:** estuary, fragmentation, nestedness, Magellan region, Pacific Ocean, Tanaidacea, Isopoda, Amphipoda

## Abstract

**Background:**

The intricate geomorphology of the coastline in the Chilean Channels and Fjords region, together with the freshwater inputs from the ice fields provide the area with very unique ecological characteristics and a variety of habitats that favour great marine biodiversity. However, although Chilean Patagonia has been the focus of several expeditions and ecological surveys, the greatest emphasis has been either on the populated coasts of the Beagle Channel and the Straits of Magellan to the south or the area to the north of Golfo de Penas, leaving vast areas that remain largely unexplored. This leads to a latitudinal gap in the faunistic information and hinders zoogeographic studies to assess biogeographical connections along the eastern coasts of the Pacific. Peracarida is a taxonomic group that provides an excellent model for such studies because of their high abundance and biodiversity, benthic habits, small size and limited dispersal capacity.

**New information:**

A dataset providing the first and only records of the benthic Peracarida between the latitudes 48–51.5°S of the Pacific coast of Chile is presented here, hence closing a geospatial gap for the study of the biogeographical connections of the Peracarida along the Eastern Pacific coast. The dataset comprises a total of 141 georeferenced records of 60 sublittoral species of Tanaidacea, Isopoda and Amphipoda. This and other studies reveal that the coastal fauna of the region follow a latitudinal distribution pattern at a larger scale and nested assemblages inside the channels and fjords that can be regarded as a consequence of the more restrictive conditions in the inner parts. In the present scenario of global warming that is expected to affect particularly polar and subpolar regions, the present dataset serves as a reference for the distribution patterns of benthic organisms with low dispersal capacity.

## Introduction

The Patagonian Channels and Fjords constitute one of the largest estuarine systems of the world, extending along around 84,000 km of coastline (Silva and Palma 2008). They receive important freshwater inputs from glaciers and pluviosity, that, together with seasonal variations in temperature, cause hydrographic gradients in salinity and temperature from the inner parts to the open ocean (Chuecas and Ahumada 1980). It is an area of highly differentiated, fragmented ecosystems that offers a unique opportunity for studying the distribution patterns of the fauna in habitats with environmental gradients.

While the southern channels and fjords of the Magellan Region have been the focus of several expeditions and ecological surveys, the greatest emphasis has been on the populated coasts of the Beagle Channel and the Straits of Magellan (De Broyer et al. 2007): for example, the scientific expedition of the *HMS Challenger* in 1873-1876 and the *Lund* expedition (Brattström and Johanssen 2011), the more recent joint Chilean-German-Italian campaign *Victor Hensen* (Arntz and Gorny 1996) and periodic sampling efforts carried out by the Universidad de Magallanes (Ríos et al. 2007). By contrast, the coastal ecosystems of the less accessible area between the Golfo de Penas and the Smith Channel (48–52°S) remain largely unexplored. The sedimentary bottoms were surveyed by the scientific expedition CIIMAR Fiordo 2 (Silva and Palma 2008, Palma and Silva 2008) from which general results for the macrofauna were presented by Mutschke (2008), who stressed the scarcity of knowledge of the amphipod fauna in the region; species lists and biogeographic remarks have been carried out for the Polychaeta (Montiel et al. 2001) and Crustacea Decapoda (Retamal and Arias 2000). On the other hand, the hard substrates of this central zone had never been surveyed until the survey carried out in Bernardo O’Higgins National Park (henceforward BONP) on-board the R/V *Nueva Galicia* (Aldea et al. 2011, Aravena et al. 2018, Esquete and Aldea 2015, Esquete et al. 2012, Palacios 2018) and whose data pertaining to peracarid crustaceans are presented here.

The superorder Peracarida is one of the most abundant and diverse taxa in the marine benthos (Cartes et al. 2001, González et al. 2008, Spears et al. 2005). Perhaps the most significant synapomorphy that characterises the group is the presence of a marsupium where the larval stages develop, with the consequent absence of a dispersive phase (Johnson et al. 2001). The displacement capacity of the adults varies across orders, families and species, with proven consequences on species geographical distribution (e.g. Bober et al. 2018). This makes the Peracarida an excellent model taxon for hypothesis testing in biogeographical research; however, they are rarely the focus of such studies: biogeographic studies of the Peracarida of the coasts of Southeast Pacific do not generally include the Magellan Region. The distribution of algae-associated Peracarida along the Chilean coasts from the northern limit down to 42°S was studied by Thiel (2002). His work revealed a latitudinal pattern, with two well-differentiated main distribution areas separated by an extensive transitional zone. Later, Thiel et al. (2003) assessed the diversity and distribution of the peracarids of Chile including the Magellan Region and Antarctic territory, highlighting the contrast between the high species richness and scarcity of data for many taxa. More recently, González et al. (2008) compiled a list of the Chilean Peracarida and their latitudinal distribution, pointing out the scarcity of records in certain latitudes and a high level of endemism, with few species with broad latitudinal ranges. Every new survey comes with the discovery of undescribed species (see, for example, Esquete et al. 2012, Esquete and Aldea 2015) showing that, despite the efforts of classic expeditions and recent surveys,knowledge of the Peracarid fauna in the area is still in its infancy.

The present dataset includes records of Peracarida along four degrees of latitude where the underwater biodiversity remained unexplored. It provides data that allow us to link the biogeography of the southern Patagonian coast with the rest of the Eastern Pacific. Additionally, the present work contributes to the knowledge of the biodiversity of the Magellan Region. All in all, the relevance of the present dataset lies in three main factors:

1. the importance of the Peracarida for biogeographic studies, due to their diversity and direct development;
2. the geomorphologic and ecological particularities of the area covered; and
3. the fact that it closes a latitudinal gap in the data available for this taxonomic group along the coasts of the eastern Pacific Ocean.

The potential of the usage of this dataset is exemplified here with nestedness analysis. It describes the species composition patterns within a continental biota and in isolated areas such as fragmented habitats and islands (Ulrich et al. 2009), which would represent a particular case of beta diversity (Ulrich and Almeida-Neto 2012). As such, nestedness constitutes an excellent means for assessing spatial patterns in terms of composition and distribution patterns in isolated ecosystems (Atmar and Patterson 1993, Escalante Espinosa and Morrone 2001).

Nestedness analysis was performed following Marin and Delgado (2001), based on the matrix of presence and absence of species by sampling site. Using the Nestcalc software (Atmar and Patterson 1993), the temperature of the observed matrix was calculated, which could vary in a range of 0-100°. A value T = 0° would correspond to a perfectly nested pattern, while T = 100° would correspond to a pattern where the set of species would be totally random (Atmar and Patterson 1993). In addition, the simulated temperature was calculated, under conditions of equal probability and Monte Carlo correction, applying 1000 iterations. The nesting graphic was obtained by the NeD software (Strona et al. 2014).

## Project description

### Title

Peracarida of Bernardo O’Higgins National Park (S Chile)

### Personnel

Cristian Aldea, Aravena Juan Carlos

### Study area description

The Bernardo O´Higgins National Park (BONP) is the largest protected area in the Southern Hemisphere with 3,525,901 hectares; its area includes the continental and archipelagic areas which extend from 47°55'S to 51°37'S.

### Funding

Chilean Production Development Corporation (CORFO)

## Sampling methods

### Study extent

**Figure 1.**
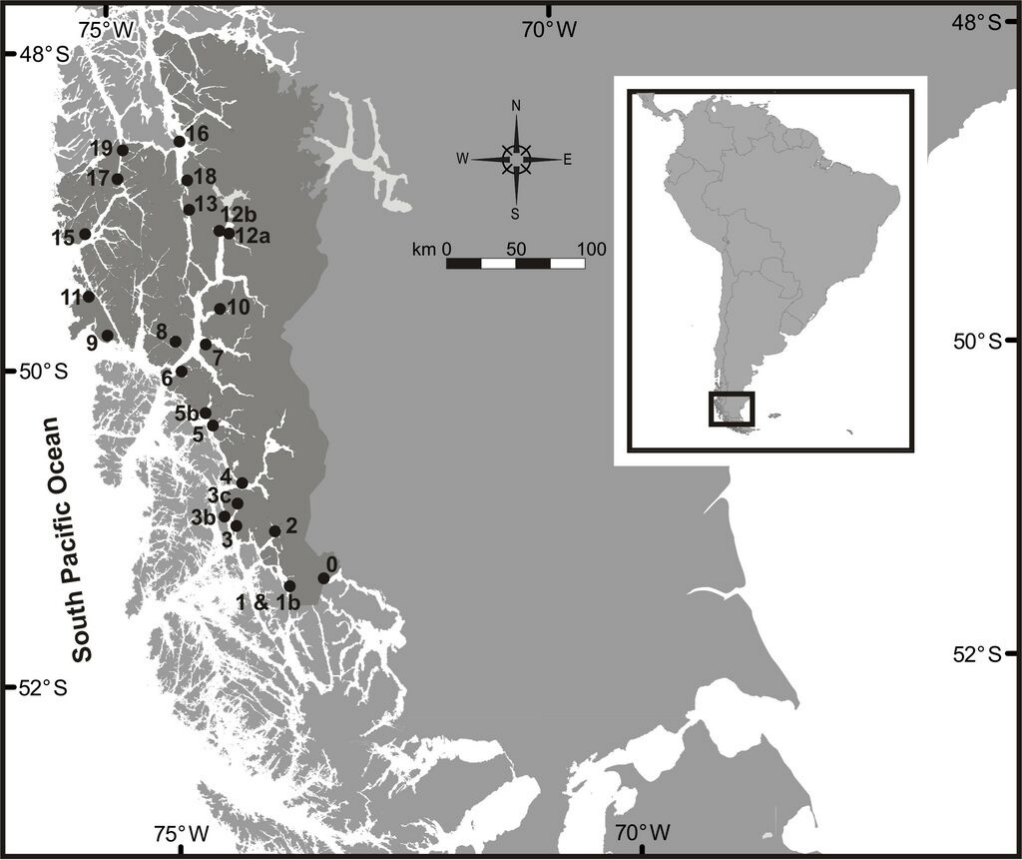
Study area with the 23 sampling sites in the BONP, Chilean Fjords and Channels Ecoregion. Dark grey area corresponds to BONP.

Sampling was performed as part of an exploratory study of the biodiversity of Bernardo O’Higgins National Park (henceforward BONP, Fig. 1). It is placed in the Chilean geopolitical regions of Aysén and Magallanes, extending along three degrees of latitude between 48.0–51.6°S and 73.3–75.8°W, in the central part of the Chilean Fjords and Channels Ecoregion (Spalding et al. 2007) and adjacent to the Southern Ice Fields. The intricate geomorphology of the coastline, together with the freshwater inputs from the ice fields provide the area with very unique ecological characteristics and a variety of habitats that favour great marine biodiversity (Aravena et al. 2018). The rocky bottoms inside the channels and fjords host abundant and diverse macroalgae, which, in turn, provide a variety of microhabitats for benthic fauna. Moreover, Palacios (2018) reported the presence of extensive kelp forests dominated by *Macrocystis pyrifera* and with the presence of *Durvillaea antarctica*, which are identified as keystone species, playing an important ecological role by providing structural support (Miller et al. 2018, Mills 2003) and enhancing dispersal (López et al. 2018) of benthic invertebrates.

### Sampling description

The rocky sublittoral bottoms of the Channels and Fjords of the BONP were sampled between January and March 2010, during two cruises on board the vessel *MV Nueva Galicia* with the objective of characterising and mapping the benthic communities.

A total of 23 sites were sampled by SCUBA divers (Fig. 1). At each site, five replicate squares of 25 × 25 cm (0.063 m2) were scraped off all the organisms, including fauna and smaller algae. Kelps were excluded from the sample and left intact on the substrate. Two samples were taken at both 5 and 15 m depth at each site (totalling 10 squares per site). Samples were fixed in 5% buffered formalin and subsequently sorted, preserved in 70% alcohol and finally the organisms identified to the lowest possible taxonomic level.

### Quality control

The records of species and their respective geographical positions of the sites were entered into a spreadsheet structured with the Darwin Core Standard (Wieczorek et al. 2012) adjusted taxonomically according to the World Register of Marine Species (WoRMS 2020). Data were submitted in the Integrated Publishing Toolkit, following standards of the Global Biodiversity Information Facility (GBIF).

## Geographic coverage

### Description

The coast of channels and fjords of the South East Pacific along three degrees of latitude.

### Coordinates

-51.521 and -48.675 Latitude; -75.392 and -73.251 Longitude.

## Taxonomic coverage

### Description

The Peracarida identified to the lowest possible taxonomic level.

### Taxa included

**Table:** 

Rank	Scientific Name	
phylum	Arthropoda	
subphylum	Crustacea	
class	Malacostraca	
order	Amphipoda	
family	Amphilochidae	
family	Ampithoidae	
family	Aoridae	
family	Atylidae	
family	Calliopiidae	
family	Colomastigidae	
family	Corophiidae	
family	Dexaminidae	
family	Eusiridae	
family	Hyalidae	
family	Iphimediidae	
family	Ischyroceridae	
family	Leptocheliidae	
family	Leucothoidae	
family	Liljeborgiidae	
family	Lysianassidae	
family	Pardaliscidae	
family	Photidae	
family	Phoxocephalidae	
family	Pontogeneiidae	
family	Stegocephalidae	
family	Stenothoidae	
family	Synopiidae	
family	Talitridae	
family	Tryphosidae	
family	Uristidae	
order	Isopoda	
family	Chaetiliidae	
family	Janiridae	
family	Spaheromatidae	
family	Stenetriidae	
order	Tanaidacea	
family	Nototaidae	
family	Tanaididae	

## Traits coverage

### Data coverage of traits

PLEASE FILL IN TRAIT INFORMATION HERE

## Temporal coverage

### Notes

2010-01-26 through 2010-03-24

## Usage rights

### Use license

Creative Commons Public Domain Waiver (CC-Zero)

### IP rights notes

This work is licensed under a Creative Commons Attribution Non Commercial (CC-BY-NC) 4.0 Licence

## Data resources

### Data package title

Peracarida of Bernardo O’Higgins National Park (S Chile)

### Resource link

https://www.gbif.org/dataset/2401cd5e-26a8-4a0c-9007-e5e6137a1364

### Alternative identifiers

http://gbif-chile.mma.gob.cl/ipt/resource?r=peracarida-pnbo

### Number of data sets

1

### Data set 1.

#### Data set name

Peracarida of Bernardo O’Higgins National Park (S Chile)

#### Data format

Darwin Core

#### Number of columns

38

#### Download URL

https://www.gbif.org/dataset/2401cd5e-26a8-4a0c-9007-e5e6137a1364#dataDescription

#### Description

A total of 60 peracarid species were identified in the area, making up a total of 141 georeferenced records (Aldea and Esquete 2020). Amphipoda were the most diverse, representing 85% (52 species), followed by the Isopoda (10%, 6 species) and the Tanaidacea (8%, 5 species).

**Data set 1.** 

Column label	Column description
id	stable identifier assigned by GBIF
institutionCode	code for the institution the record belongs to
collectionCode	code of the physical collection within the institution
basisOfRecord	observation type
occurrenceID	unique identifier of the occurrence
recordedBy	name of the person responsible for the record
individualCount	number of specimens
preparations	preservation/storage method
eventDate	date of the event
catalogNumber	identifier within the physical collection
scientificNameAuthorship	authorship information for the scientific name formatted according to the conventions of the applicable nomenclaturalCode
geodeticDatum	the ellipsoid, geodetic datum or spatial reference system (SRS) upon which the geographic coordinates given are based
habitat	habitat of the occurrence
samplingProtocol	collection methods
countryCode	code of the country of the occurrence location
stateProvince	province and region of the occurrence location
county	county or commune of the occurrence location
locality	locality of the occurrence location
decimalLatitude	latitude in decimal degrees for the occurrence location
decimalLongitude	longitude in decimal degrees for the occurrence location
verbatimCoordinateSystem	coordinate system as originally indicated
verbatimSRS	spatial reference system as originally indicated
identificationQualifier	identification qualifier for the taxon
identifiedBy	name of the person/s who identified the occurrence
dateIdentified	date when the occurrence was identified
scientificName	scientific name of the lowest taxonomic level attained for the occurrence. If species level, full scientific name, with authorship and date information
kingdom	kingdom of the occurrence
phylum	phylum of the occurrence
class	class of the occurrence
order	order of the occurrence
family	family of the occurrence
genus	genus of the occurrence
specificEpithet	species name of the occurrence
taxonRank	lowest taxon rank of identification of the occurrence
verbatimLatitude	verbatim original latitude of the Location
verbatimLongitude	verbatim original longitude of the Location
country	country of the occurrence
coordinateUncertaintyInMetres	horizontal distance (in metres) from the given decimalLatitude and decimalLongitude describing the smallest circle containing the whole of the Location

## Additional information

**Figure 2.**
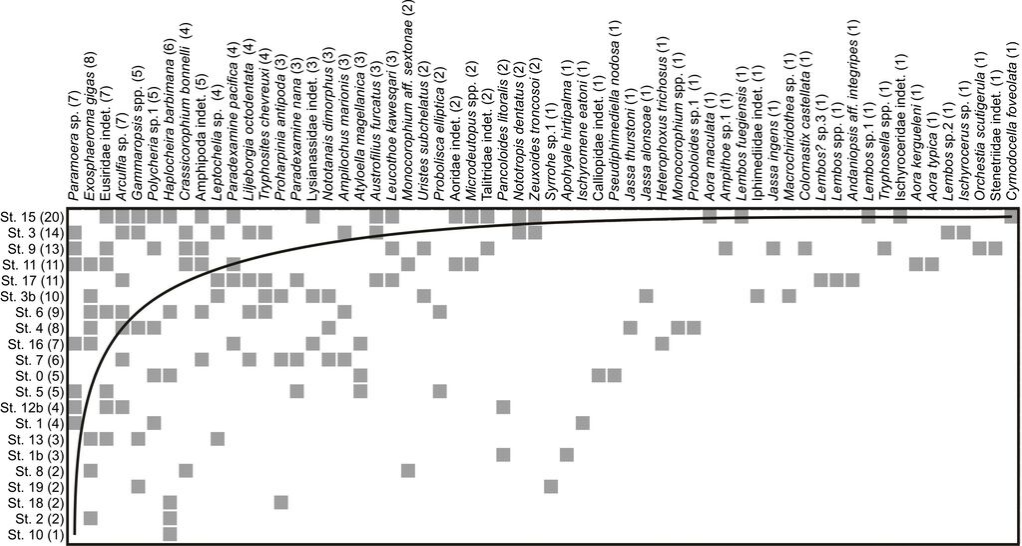
Analysis of nestedness of peracarids of the BONP. Species presence is marked with a grey square, absence with white. The lines of perfect order (minimum temperature) are indicated in the matrix. Vertical axis: sampling sites (St), number of species in brackets. Horizontal axis: Species found, with the number of sites where the species was found in brackets.

The results showed that the Peracarida of the BONP presented a nested pattern, with a larger number of species in the outermost part of the channels and subsets of those in the innermost. The temperature of the matrix was 20.762°C; p < 0.01 (Fig. 2).

This study widens the known distribution limit of several species previously recorded in Patagonia and the Southern Ocean (compiled in De Broyer et al. 2007, Horton et al. 2020): for instance, the amphipod *Aora maculata* (Thomson, 1879) has been recorded in several locations throughout the Southern Ocean and this is the first record in the American continent. Likewise, the amphipods *Jassa ingens* (Pfeffer, 1888), *Jassa thurstoni* Conlan, 1990 and *Heterophoxus trichosus* K.H. Barnard, 1932 and the isopod *Ischyromene eatoni* (Miers, 1875), whose distributions were previously confined to the Scotia Arch Islands and the Southern Ocean, are registered for the first time on this latitude. *Colomastix castellata* K.H. Barnard, 1932 was described from Islas Malvinas (Falkland Islands) and is found for the first time since the original description. *Uristes subchelatus* (Schellenberg, 1931) is found for the first time out of the Magellan Strait. A total of 50% of the species occurred in one site only, which can be interpreted as a consequence of the heterogeneity and habitat fragmentation of the area and the niche specificity of Peracarid species.

Generally, species distribution patterns observed at the present time are the result of several ecological, evolutionary and biological processes. Given that nested patterns can be the result of extinction and/or colonisation processes (González and Poulin 2005), the results presented here have three possible interpretations:

1. as a colonisation process from the open ocean towards the inner parts of the channel;
2. as a consequence of an increased heterogeneity and hence the higher number of available niches towards the outer part of the channels; or
3. as a consequence of a gradient of environmental conditions along the channels being less favourable to the development of the Peracarids towards the inner part.

Nested patterns have been found previously in the marine fauna of the region, generally consistent with the latitudinal gradient: fish parasite species (González and Poulin 2005) and polychaete assemblages (Moreno et al. 2006) adjust to this pattern, in both cases revealing that the majority of the species find their optimum at a certain range of latitude. Perhaps more interestingly, a biogeographical study of the calanoid copepods of the southern Chilean channels showed a nested pattern characterising various subsets of a larger assemblage corresponding to inner “microbasins” within the channels (Marin and Delgado 2001).

### Conclusions

The combination of the results presented here and those of the mentioned previous studies seems to indicate that, whereas at a larger scale, there are clear latitudinal patterns in the distribution of the marine species along the southeast Pacific coasts, the distribution on a finer scale responds to more specific ecological preferences of the species and the more extreme environmental conditions in the inner part of channels and fjords.

This dataset compiles the first and only records of the benthic Peracarida in the channels and fjords of the Pacific coast of Chile between the 48–51.5°S, hence closing a latitudinal gap for the study of the biogeographical connections of the group along the Eastern Pacific coast. In the present scenario of global warming that is expected to affect particularly polar and subpolar regions, the present dataset serves as a reference for the distribution patterns of benthic organisms with low dispersal capacity.

## References

[B6068894] Aldea C, Césped T, Rosenfield S (2011). Opistobranchs from Bernardo O'Higgins National Park (S. Chile). Thalassas.

[B6077217] Aldea C, Esquete P (2020). Peracarida of Bernardo O’Higgins National Park (S Chile). Version 1.2. Occurrence dataset.

[B6068903] Aravena Juan-Carlos, Vela-Ruiz Germaynee, Torres Juliana, Huenucoy Carolina, Tonko Juan-Carlos (2018). Parque nacional bernardo o’higgins/territorio kawésqar waes: Conservación y gestión en un territorio ancestral. Magallania (Punta Arenas).

[B6068913] Arntz W, Gorny M (1996). Cruise report of the Joint Chilean-German-Italian Magellan" Victor Hensen" Campaign in 1994. Berichte zur Polarforschung.

[B6068922] Atmar Wirt, Patterson Bruce D. (1993). The measure of order and disorder in the distribution of species in fragmented habitat. Oecologia.

[B6068931] Bober Simon, Brix Saskia, Riehl Torben, Schwentner Martin, Brandt Angelika (2018). Does the Mid-Atlantic Ridge affect the distribution of abyssal benthic crustaceans across the Atlantic Ocean?. Deep Sea Research Part II: Topical Studies in Oceanography.

[B6068959] Brattström Hans, Johanssen Arild (2011). Ecological and regional zoogeography of the marine benthic fauna of Chile. Sarsia.

[B6115617] Cartes J. E, Elizalde M, Sorbe J. C (2001). Contrasting life-histories, secondary production, and trophic structure of Peracarid assemblages of the bathyal suprabenthos from the Bay of Biscay (NE Atlantic) and the Catalan Sea (NW Mediterranean). Deep Sea Research Part I: Oceanographic Research Papers.

[B6068968] Chuecas Lisandro M, Ahumada Ramon B (1980). Contribucion al conocimiento de las condiciones hidrograficas de los fiordos de la region magallanica - Chile. Boletim do Instituto Oceanográfico.

[B6068977] De Broyer C, Lowry J K, Jażdżewski K, Robert H, De Broyer C (2007). Catalogue of the Gammaridean and Corophiidean Amphipoda (Crustacea) of the Southern Ocean, with distribution and ecological data. Census of Antarctic Marine Life: Synopsis of the Amphipoda of the Southern Ocean.

[B6068999] Escalante Espinosa T, Morrone J J, Llorente Bousquets J, Morrone J J (2001). Para qué sirve el análisis de parsimonia de endemismos?. Introducción a la biogeografía en Latinoamérica: teorías, conceptos, métodos y aplicaciones.

[B6069012] Esquete Patricia, Bamber Roger, Aldea C (2012). On some shallow-water Tanaidomorpha (Crustacea: Peracarida: Tanaidacea) of Chilean fjords, with description of a new species of Zeuxoides Sieg, 1980. Zootaxa.

[B6069021] Esquete Patricia, Aldea Cristian (2015). *Leucothoe
kawesqari*, a new amphipod from Bernardo O’Higgins National Park (Chile), with remarks on the genus in the Magellan Region (Crustacea, Peracarida). ZooKeys.

[B6069030] González Exequiel R, Haye Pilar A, Balanda Maria-José, Thiel Martin (2008). Lista sistemática de especies de peracáridos de Chile (Crustacea, Eumalacostraca). Gayana (Concepción).

[B6069039] González M. T., Poulin R. (2005). Nested patterns in parasite component communities of a marine fish along its latitudinal range on the Pacific coast of South America. Parasitology.

[B6069048] Horton T, Lowry J, De Broyer C, Bellan-Santini D, Coleman C O, Corbari L, Costello m J, Danelilla M, Dauvin J-C, Fišer C, Gasca R, Grabowski M, Guerra-Garcia J M, Hendrycks E, Hughes L, Jaume D, Jazdzewski K, Kim Y-H, King R, Krapp-Schickel T, LeCroy S, Lörz A-N, Mamos T, Senna A R, Serejo C, Sket B, Souza-Filho J F, Tandberg A H, Thomas J D, Thurston M, Vader W, Väinölä R, Vonk R, White K, Zeider W World Amphipoda Database. http://www.marinespecies.org/amphipoda/.

[B6114769] Johnson William S., Stevens Margaret, Watling Les (2001). Reproduction and development of marine peracaridans. Advances in Marine Biology.

[B6069106] López Boris A., Macaya Erasmo C., Rivadeneira Marcelo M., Tala Fadia, Tellier Florence, Thiel Martin (2018). Epibiont communities on stranded kelp rafts of *Durvillaea
antarctica* (Fucales, Phaeophyceae)-Do positive interactions facilitate range extensions?. Journal of Biogeography.

[B6069131] Marin L H, Delgado L E (2001). The taxocenosis of calanoid copepods in the magellan Inlets: a nested pattern. Ciencia y Tecnología del Mar.

[B6069140] Miller Robert J., Lafferty Kevin D., Lamy Thomas, Kui Li, Rassweiler Andrew, Reed Daniel C. (2018). Giant kelp, *Macrocystis
pyrifera*, increases faunal diversity through physical engineering. Proceedings of the Royal Society B: Biological Sciences.

[B6069151] Mills Eric L. (2003). Deep-sea Amphipoda from the Western North Atlantic Ocean. The Family Ampeliscidae. Limnology and Oceanography.

[B6069169] Montiel A, Rios C, Mutschke E, Rozbaczylo N (2001). Poliquetos de fiordos y canales adyacentes al campo de hielo patagónico sur, Chile (Annelida, Polychaeta). Ciencia y Tecnología del Mar.

[B6069178] Moreno Rodrigo A., Hernandez Cristian E., Rivadeneira Marcelo M., Vidal Marcela A., Rozbaczylo Nicolas (2006). Patterns of endemism in south-eastern Pacific benthic polychaetes of the Chilean coast. Journal of Biogeography.

[B6069188] Mutschke E, Silva N, Palma S (2008). Macrobenthic biodiversity and community structure in austral Chilean channels and fjords. Progress in the oceanographic knowledge of chilean interior waters, from Puerto Montt to Cape hornos.

[B6069201] Palacios Mauricio (2018). Macroalgas submareales de los canales interiores del Parque Nacional Bernardo O’Higgins (~49°- 51° S), región de Magallanes, Chile. Anales del Instituto de la Patagonia.

[B6069210] Palma S, Silva N, Palma S, Silva N (2008). Scientific results of the CIMAR Program in the austral Chilean channels and fjords. CIMAR 1 to 4 Fiordos cruises. Progress in the oceanographic knowledge of Chilean interior waters, from Puerto Montt to Cape Horn.

[B6069223] Retamal M A, Arias A A (2000). Análisis cualitativo de los decápodos recolectados en la región de fiordos y canales (entre Golfo de Penas y Estrecho de Magallanes) (CIMAR-FIORDOS 2). Ciencia y Tecnología del Mar.

[B6069232] Ríos Carlos, Arntz Wolf E., Gerdes Dieter, Mutschke Erika, Montiel Américo (2007). Spatial and temporal variability of the benthic assemblages associated to the holdfasts of the kelp Macrocystis
pyrifera in the Straits of Magellan, Chile. Polar Biology.

[B6069242] Silva N, Palma S, Silva N, Palma S (2008). The CIMAR Program in the austral Chilean channels and fjords. Progress in the oceanographic knowledge of Chilean interior waters, from Puerto Montt to Cape Horn.

[B6069255] Spalding Mark D., Fox Helen E., Allen Gerald R., Davidson Nick, Ferdaña Zach A., Finlayson Max, Halpern Benjamin S., Jorge Miguel A., Lombana Al, Lourie Sara A., Martin Kirsten D., McManus Edmund, Molnar Jennifer, Recchia Cheri A., Robertson James (2007). Marine ecoregions of the World: A bioregionalization of coastal and shelf areas. BioScience.

[B6115626] Spears Trisha, DeBry Ronald W., Abele Lawrence G., Chodyla Katarzyna (2005). Peracarid monophyly and interordinal phylogeny inferred from nuclear small-subunit ribosomal DNA sequences (Crustacea: Malacostraca: Peracarida). Proceedings of the Biological Society of Washington.

[B6069275] Strona Giovanni, Galli Paolo, Seveso Davide, Montano Simone, Fattorini Simone (2014). Nestedness for dummies (NeD): A user-friendly web interface for exploratory nestedness analysis. Journal of Statistical Software.

[B6069285] Thiel Martin (2002). The zoogeography of algae-associated peracarids along the Pacific coast of Chile. Journal of Biogeography.

[B6069294] Thiel M, González E R, Balanda P, Haye R, Heard R, Watling L, Hendrickx M E (2003). Diversity of Chilean peracarids (Crustacea: Malacostraca). Contributions to the Study of East Pacific Crustaceans 2.

[B6069309] Ulrich Werner, Almeida-Neto Mário, Gotelli Nicholas J. (2009). A consumer's guide to nestedness analysis. Oikos.

[B6069318] Ulrich Werner, Almeida-Neto Mário (2012). On the meanings of nestedness: back to the basics. Ecography.

[B6069327] Wieczorek John, Bloom David, Guralnick Robert, Blum Stan, Döring Markus, Giovanni Renato, Robertson Tim, Vieglais David (2012). Darwin Core: An evolving community-developed biodiversity data standard. PLOS One.

[B6069340] WoRMS Peracarida. http://www.marinespecies.org/aphia.php?p=taxdetails&id=1090.

